# Characterization of acute myocardial infarction by pre-contrast T1 mapping

**DOI:** 10.1186/1532-429X-14-S1-P28

**Published:** 2012-02-01

**Authors:** Erica Dall'Armellina, Stefan K Piechnik, Vanessa Ferreira, Jane M Francis, Matthew D Robson, Florim Cuculi, Rajesh Kharbanda, Adrian P Banning, Robin P Choudhury, Theodoros Karamitsos, Stefan Neubauer

**Affiliations:** 1 Department of Cardiovascular Medicine, University of Oxford Centre for Clinical Magnetic Resonance Research, Oxford, UK; 2 Department of Cardiology, John Radcliffe Hospital, Oxford, UK

## Summary

Novel CMR techniques are needed to assess reversible myocardial injury in acute MI. Our results show that pre-contrast T1 mapping CMR could be such a technique: increasingly higher T1 values are associated with larger extent of acute myocardial injury and with reduced functional recovery at 6 months.

## Background

Cardiovascular magnetic resonance (CMR) techniques such as late gadolinium enhancement (LGE) and edema imaging (T2W) are used to delineate acute myocardial infarction (MI). However, the use of LGE and T2W to assess reversible injury acutely is challenged by the dynamic changes occurring in the myocardial tissue. T1-mapping is a novel technique that provides voxel-wise quantitative information on the regional tissue state and therefore can characterize in detail the various components of ischemic injury. In acute MI patients, we sought to investigate whether pre-contrast T1-mapping11 (1) detects acute myocardial injury, (2) allows for quantification of the severity of damage when compared to standard techniques such as LGE and T2W, and (3) has the ability to predict long term functional recovery.

## Methods

3T CMR including T2W, T1 mapping and LGE was performed in patients with acute MI at 12-48 hour after chest pain onset and at 6 months. Patients with ST elevation MI (STEMI) underwent primary PCI prior to CMR. Assessment of acute regional wall motion abnormalities, acute segmental damaged fraction by T2W and LGE and mean segmental T1 values was performed on matching short axis slices. LGE and improvement in regional wall motion at 6M were also obtained.

## Results

**Figure 1:**
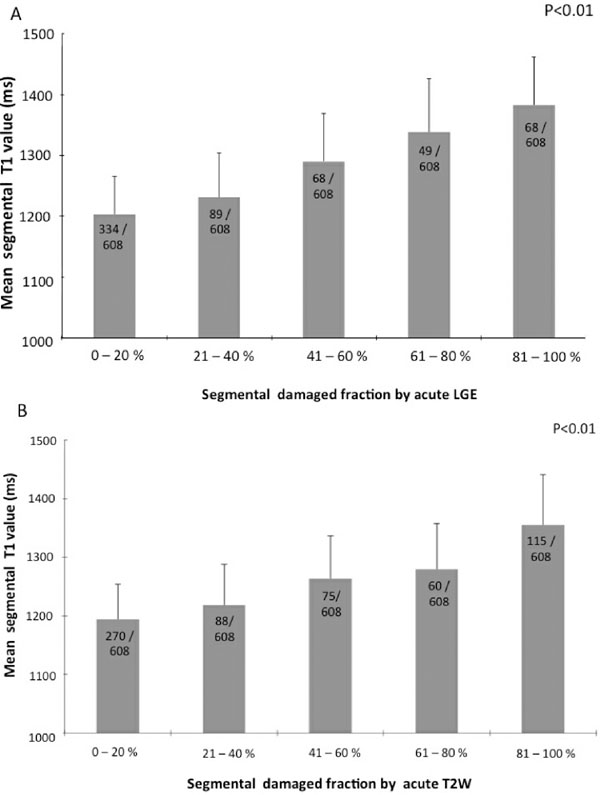


**Figure 2:**
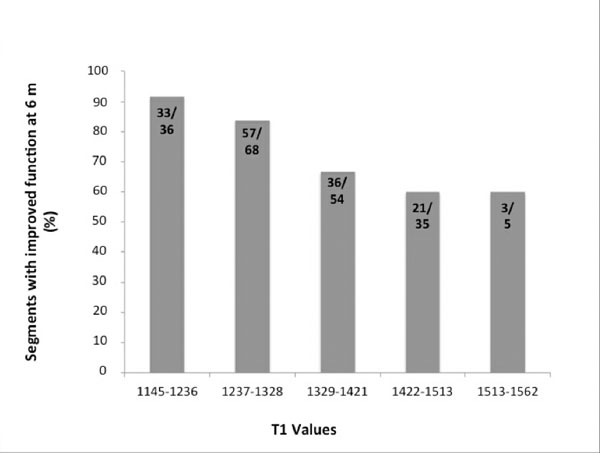


High T1 values were shown in acutely injured segments by LGE and T2W. The diagnostic performance of acute T1-mapping was at least as good as that of T2W CMR for detecting myocardial injury. We found increasingly significantly higher T1 values in segments with larger damaged fraction assessed by either by LGE or T2W (P<0.01) (Fig.1). The index of salvaged myocardium derived by acute T1 mapping and 6M LGE was not different from the one derived from T2W (P=0.88). Furthermore, the likelihood of improvement of segmental function at 6 months decreased progressively as acute T1 values increased (P<0.0004) (Fig.2).

## Conclusions

In patients with acute MI, incremental increases in pre-contrast T1 values delineate the extent of myocardial injury and predict functional recovery at 6 months. T1 mapping might become an important complementary technique to LGE and T2W for characterization of acute myocardial injury.

## Funding

Oxford Comprehensive Biomedical Research Centre, NIHR funding scheme.

